# Deprescribing in palliative patients with cancer: a concise review of tools and guidelines

**DOI:** 10.1007/s00520-021-06605-y

**Published:** 2021-10-07

**Authors:** Lisanne N. van Merendonk, Mirjam Crul

**Affiliations:** 1 Department of Pharmacy & Pharmacology, The Netherlands Cancer Institute-Antoni Van Leeuwenhoek, Plesmanlaan 121, 1066 CX Amsterdam, The Netherlands; 2 Department of Clinical Pharmacy and Pharmacology, Amsterdam UMC, VU Medical Center, Amsterdam, The Netherlands

**Keywords:** Cancer, Deprescribing, Palliative care, PIMs

## Abstract

**Purpose:**

Palliative cancer patients can benefit from deprescribing of potentially inappropriate medications (PIMs). Tools and guidelines developed for the geriatric population are mainly available. This systematic review gives an overview of available guidelines and tools to deprescribe for palliative cancer patients.

**Methods:**

A systematic search was carried out using the databases SCOPUS and PubMed. Studies focused on palliative cancer patients were included.

**Results:**

The search identified 137 studies of which 15 studies were included in this systematic review. Six of the included tools were developed specifically for cancer patients. One of these tools was externally validated and applied in several studies and settings. Guidelines or tools that were not specifically developed for cancer patients but that were applied on cohorts of palliative cancer patients were also included.

**Conclusion:**

Tools developed for geriatric patients contain drugs that are not inappropriate when used in the palliative cancer care setting. Tools developed for cancer patients are more suitable and can be applied in combination with stepwise methods to individualize deprescribing per patient. The tools and guidelines described in this systematic review can be used to further implement deprescribing in the clinical routine for palliative cancer patients.

## Introduction

The use of a multitude of drugs is often termed “polypharmacy.” In general, most practitioners understand this term in a negative connotation, when patients are taking a relatively large number of different drugs, and/or a number of drugs for which the appropriateness may be questionable (potentially inappropriate medications (PIMs)). Polypharmacy has been associated with an increased risk for adverse events, a higher symptom burden, and a lower quality of life [1, 2]. Especially patients with cancer can be prescribed many drugs: systemic anti-cancer treatments, often combined with supportive drugs, and additional treatments to decrease symptom burden or alleviate side effects. Since cancer is a disease of which the incidence increases with age, many patients also suffer from one or more comorbidities for which drugs are in use. More often than not, this results in complex drug schemes that patients or their caregivers have to manage. Patients with cancer may transition at some point from being on a treatment with curative intent to palliative care. This transition is often accompanied by a limited life expectancy. At this point, the benefit-risk ratio of medications can shift, for example, in the case of drugs prescribed as primary prophylaxis. The occurrence of polypharmacy and the use of potentially inappropriate medications (PIMs) in patients with advanced cancer have been studied and described in several publications. The largest study researched a patient cohort from Sweden, including > 150,000 individuals, and found that in the last month of life 60% of patients were on continued antihypertensive treatment, 17% on lipid-lowering drugs, and 19% on mineral supplements [3]. A review from 2014 identified 9 trials that examined the use of PIMs in palliative cancer patients, yielding percentages of patients that receive one or more PIMs from 22 up to 95%, depending on the study population as well as on the criteria used to determine which drugs can be regarded as PIMs [4].

The concept of deprescribing, to reduce polypharmacy and/or the number of PIMs in patients, was defined by Scott et al. as: “a systematic process of identifying and discontinuing drugs in instances in which existing or potential harms outweigh existing or potential benefits within the context of an individual patients’ goals, current level of functioning, life expectancy, values and preferences” [1]. This concept serves multiple aims, for it has been shown that reducing the pill burden of patients with a limited life expectancy can increase quality of life [2, 5], decrease the risk of side effects or worse clinical outcome [6, 7], and reduce healthcare costs [3, 8, 9].

In conclusion, the benefits of deprescribing in patients with cancer in the palliative phase are well established. However, there is as yet no gold standard or consensus on a guideline or deprescribing tool to do so. In this review, we will summarize and compare the available options that have been published in the scientific literature.

## Methods

### Inclusion and exclusion criteria

We included studies when they included palliative cancer patients. Therefore, studies focusing on a population without palliative cancer patients were excluded. Studies applying a tool or certain criteria for screening of medications were included if they were applied to palliative cancer patients. All publications that included tools in palliative care settings, including those specific for cancer or not specifically developed for cancer patients, were eligible for inclusion. Studies not applying a tool or guideline were excluded. Studies focusing on one specific medication category were also excluded. Only electronic articles available in English were included.

### Search strategy

We carried out a literature search in December 2020 using the databases SCOPUS and PubMed. We searched these databases with the following terms: (pallia* OR palliative care) AND (oncology OR oncol* OR cancer OR metastat*) AND (deprescribing OR deprescribe OR deprescription OR “de-prescribing” OR “inappropriate prescribing” OR “inappropriate medications” OR “inappropriate medication” OR “unnecessary prescription” OR “unnecessary prescriptions”). One author (LM) carried out the search.

### Data processing

Two authors (LM, MC) independently screened titles and abstracts of studies retrieved by the literature search after removal of duplicates for eligibility. Afterwards, the full-text articles were assessed for inclusion by two authors (LM, MC), also independently. When the decision on inclusion yes or no differed between the two authors, an ultimate decision was reached after discussing the content of the paper. Data extraction was conducted of the included studies and summarized.

## Results

**Fig. 1:**
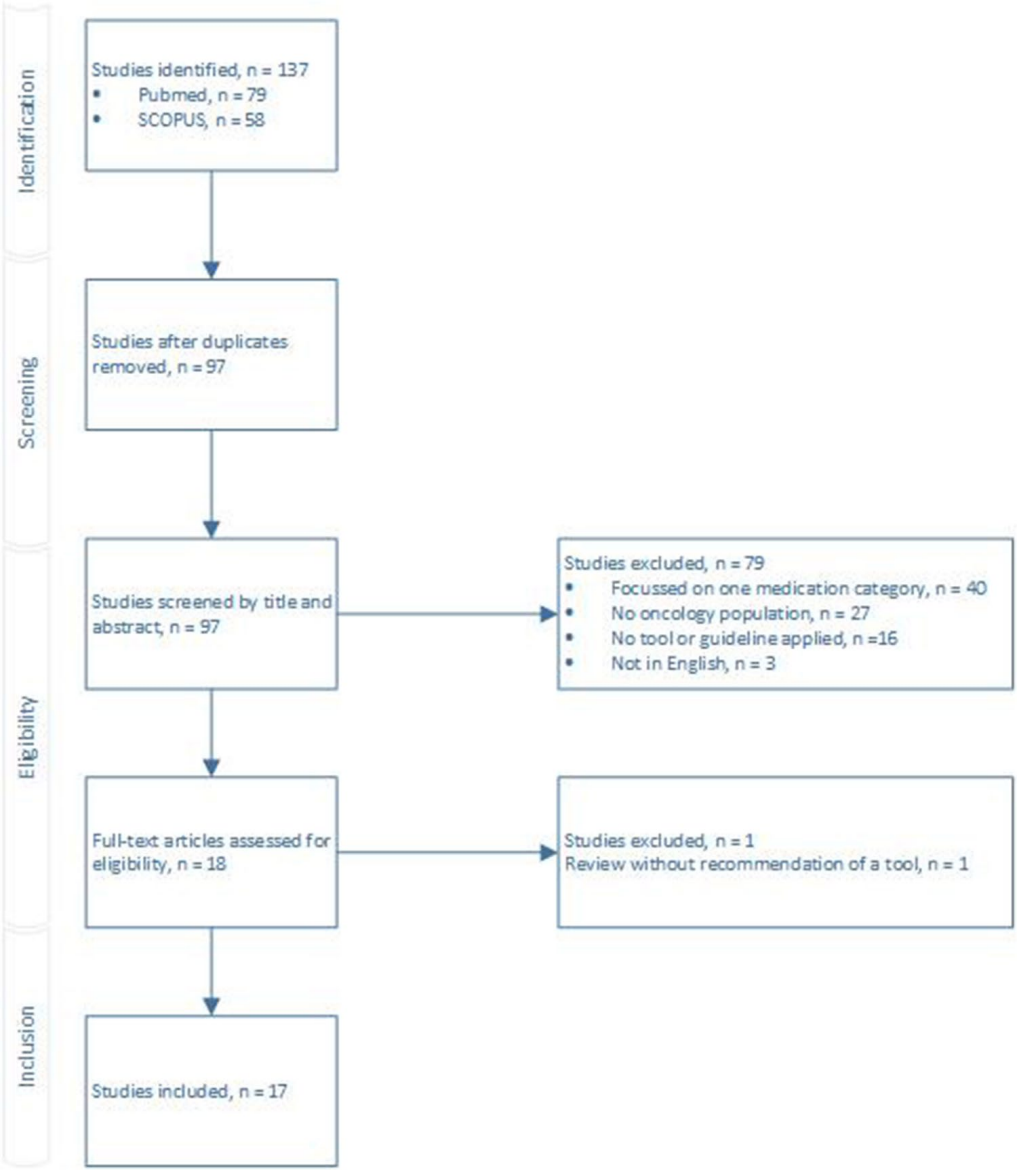
Preferred reporting item for systematic reviews and meta-analysis (PRISMA) flow diagram

The search identified 137 studies of which 17 studies were eligible for inclusion (Fig. 1). There were no discrepancies between the individual scores of the two screening pharmacists. Eight studies applied a tool not developed for the oncology population or a palliative cancer care population. In two articles, the authors recommended a methodology specific for oncology patients without applying or validating the tool. Since a few studies applied the same tool, in total, nine different tools or guidelines were identified. Table 1 summarizes these tools and guidelines and Table 2 shows the outcomes of the studies applying these tools. All identified tools and guidelines are further described below.

**Table 1:** Summary of tools and guidelines identified

Tool	Description	Target population during development
OncPal [10]	Validated against an expert opinion panel in a single-center study. It includes medications with a limited benefit in palliative cancer patients. It consists of 8 medication classes: anticoagulants, cardiovascular agents, osteoporosis medications, peptic ulcer prophylaxis, oral hypoglycemics, vitamins, minerals, and complementary-alternative medicines	Palliative cancer patients with a life expectancy < 6 months
6-Step method [11]	A systematic method for deprescribing consisting of 6 steps Step 0: reappraisal of the patient’s clinical situation, setting treatment goals Step 1: to find out all the medications a patient is taking Step 2: agreement with patient and carers Step 3: identify drugs that can be deprescribed in the first place without causing harm Step 4: address medication that requires a long time until benefit, outside of the patients’ expected lifespan Step 5: identification of medications that could be withdrawn, but slowly Step 6: monitor carefully to identify clinical problems	Advanced cancer patients
Steps to deprescribe [12]	A periodically carried out comprehensive medication assessment following 5 steps to deprescribe: Step 1: reconcile all medications and consider indications Step 2: consider overall risk of harm Step 3: assess each drugs in terms of current or future benefit in relation to current or future harm Step 4: prioritize drugs for deprescribing, giving preference to those that have the most unfavorable risk/benefit ratio and least likelihood of withdrawal symptoms Step 5: implement a discontinuation plan and monitor	Older patients with cancer
Futility criteria by Oliveira et al. [13]	Criteria for futility of 7 medication categories, criteria modified from Fede et al. [14]. Medication categories included conditions for futility. Medication categories covered gastric protectors, antihypertensive drugs, antidiabetic drugs, statins, anticoagulants, bisphosphonates, and antidementia drugs	Advanced cancer patients with a life expectancy < 6 months
Preventative medications by Todd et al. [15]	Classes of the most common inappropriate preventative medication in patients with life-limiting illness based on a systematic review: vitamins and minerals, antidiabetic, antihypertensive, antihyperlipidemic, and antiplatelet medications	Patients with a life-limiting illness
Medications for chronic diseases by Garfinkel et al. [16]	Medications for chronic diseases. Topical preparations and drugs for oncological treatments were excluded (oral and/or intravenous cytostatic drugs and biological agents)	End-stage cancer patients referred to homecare hospice
Beers criteria [17]	PIMs to avoid by older adults in most circumstances or under specific situations, updated by the American Geriatrics Society	Geriatric population
STOPP criteria [18]	Screening tool of older people’s prescription (STOPP) criteria consists of 80 criteria. These medications are associated with adverse drug events and can be used for older people	Older patients
Medication appropriateness index [19]	A questionnaire of 10 questions used by physicians to fill in a score to assess if the use of a certain drug is appropriate of inappropriate. Questions are focused on, e.g., indications, dosage, durations, interactions, and effectiveness	Ambulatory, elderly patients

**Table 2:** Outcomes of PIMs in studies applying tools or guidelines

Article	No. cancer patients	Population type	Study type	Criteria for PIM	%*n* ≥ 1 PIM	% PIMs	Most frequently prescribed PIM
Use of tool developed specific for cancer patients							
Marin et al. [20]	266	Cancer patients seen by the palliative care consult team	Retrospective database review	OncPal (not used by palliative team)	82% before consultation, 57% after consultation	21% PIMs before, 14% PIMs after	Vitamin, minerals Antihypertensives Peptic ulcer prophylaxis
Wenedy et al. [21]	6158	Cancer patients in home hospice care	Retrospective study	OncPal to assess appropriateness of discontinuation	NA	NA	Omeprazole, furosemide, simvastatin
Lindsay et al. [10]	61	Palliative cancer in patients with < 6-month prognosis	Prospective, non-interventional cohort study	OncPal	70%	21.4%	Antihypertensive, dyslipidemic agents, CAMs
Todd et al. [22]	125 in the UK 191 in the USA	Patients who died of lung cancer with a hospital admission within the last 6 months of life	Retrospective cohort study	The most common inappropriate preventative medications	At admission: 73% in the UK 80% in the USA At discharge: 63% in the UK 69% in the USA	NA	UK: antihypertensive agents US: vitamin and minerals
Oliveira et al. [13]	448 patients	Patients referred to the palliative care service of an oncology institute	Retrospective analysis	Focus on the prescription of gastric protectants, antihypertensive agents, antidiabetic agents, anticoagulants, antidementia drugs, and statins (criteria modified from Fede et al. [14])	Futility within categories: Statins: 97% Gastric protectors: 50% Antihypertensive agents: 27% Antidiabetic: 1% Bisphosphonates: 26% Antidementia: 100%	NA	Gastric protectants
Garfinkel et al. [16]	202 patients	End-stage cancer patients at the time of admission to homecare hospice	Retrospective chart review	Medications for chronic diseases, excluding oncological treatments Appropriateness of preventative medication was not assessed	NA	NA	2 months before death: 31% patients were treated with statins 23% with aspirin 16% with blood pressure-lowering drugs
Use of tool developed not specific for cancer patients							
Karuturi et al. [23]	1595 breast cancer patients 1528 colorectal cancer patients	Patients ≥ 65 years with breast or colorectal cancer receiving adjuvant chemotherapy	Retrospective cohort study	DAE and Beers criteria	NA	At baseline DAE criteria: 22.2% in the breast cohort 15.5% in the colorectal cohort Beers criteria: 27.6% in the breast cohort 24.8% in the colorectal cohort	
Karuturi et al. [24]	1595 breast cancer patients 1528 colorectal cancer patients	Patients ≥ 66 years with stage II/III breast or colorectal cancer receiving adjuvant chemotherapy	Retrospective cohort study	STOPP criteria	NA	31.5% in the breast cohort 30.9% in the colorectal cohort	NA
Hong et al. [25]	301	Older adults (≥ 70 years) with histologically diagnosed solid cancer who were candidates for first-line palliative chemotherapy	Secondary analysis of a prospective observational study	2015 Beers criteria with exclusion of medication typically used to alleviate chemotherapy-induced nausea	45.5%	12.4%	
Nightingale et al. [26]	172 patients who used no complementary and alternative medications (CAM) 62 patients who used complementary and alternative medications	Ambulatory older adults with cancer who received an initial comprehensive geriatric oncology assessment	Secondary analysis of a retrospective study	Three tools: -STOPP criteria -DAE -Beers criteria	No CAM: 52.3% CAM: 50%	NA	NA
Flood et al. [27]	47	Older adult cancer patients admitted to the oncology-acute care for elderly	Prospective, observational study	Beers criteria	21% on admission	NA	PRN promethazine for nausea Diphenhydramine before blood transfusion
Nightingale et al. [28]	142 of which 41 received iMAP	Patients ≥ 65 years who received an initial geriatric oncology assessment	Prospective, exploratory pilot into pharmacist-led individualized medication assessment and planning (iMAP)	Beers criteria	All patients: 39.4% Patient that received iMAP: 46%	NA	NA
Zhou et al. [29]	311 chemotherapy order templates	No patients were included	Review of order templates	Six medications defined as PIMs by the Beers criteria and frequently prescribed for supportive care: antihistamines, benzodiazepines, corticosteroids, H2-receptor antagonists, metoclopramide, and antipsychotics	45% of the chemotherapy order templates	NA	Antihistamines (39.5% of the templates)
Domingues et al. [19]	71 patients	Cancer patients at the time of transition to the palliative care setting	Prospective observational study	Medication appropriateness index	NA	After first consultation in the palliative care setting: 28.2% drugs were suspended	Most frequently suspended medications: Psychoactive drugs (13.5%) Analgesics (12.4%) Laxatives (9.6%)

*NA* not available

### Tools specifically for cancer patients

#### OncPal

Three of the included studies evaluated the use of the OncPal guideline specifically developed for deprescribing in palliative cancer patients. Lindsay et al. [10] describes the development and validation of this guideline against an expert opinion panel in a single-center study. The OncPal guideline was shown to match the deprescription of 617 medicines in 61 patients with an accuracy of 94% when compared to the expert panel. In the 61 patients, 70% were taking at least one PIM, and of the total medicines that were used in the patient group, 21.4% were assessed as PIMs. The OncPal guideline gives deprescribing advice on 8 classes of drugs. Two other studies also assessed PIMs using the OncPal method (Table 2). Marin et al. [20] compared PIMs prior to and after a palliative care consult in non-curative in patients with cancer. They expanded the medication categories in the OncPal with anticoagulants and benzodiazepine receptor agonists and showed that a palliative care consult could reduce the percentage of PIMs from 21 to 14% in palliative cancer patients. Wenedy et al. [21] assessed the appropriateness of discontinuation of medications in cancer and non-cancer home hospice care patients using OncPal. No absolute amounts of PIMs were measured. However, the use of most of the preventive medications was discontinued in 60 to 70% of the included patients, with proton pump inhibitors being the drug class most often de-escalated or halted.

#### 6-Step method

The 6-step method as proposed by Gonçalves [11] is described in Table 1. This is a systematic method to make deprescribing more efficient and safer. This method was suggested and described in a review. However, it gives no detailed guidance on which drugs for which indications can be safely deprescribed, nor has it been assessed in actual patients.

#### Steps to deprescribe

Also Sharma et al. [12] propose a stepwise method to deprescribe in older patients with cancer to increase appropriateness and safety during deprescribing (Table 1). These steps should be carried out periodically. This method was recommended for older patients with cancer and did not focus specifically on palliative cancer patients. Furthermore, like the 6-step method described above, this method has not been applied on a patient population.

#### Futility criteria

Oliveira et al. [13] modified criteria for futility from Fede et al. [14] into a guideline to assess futility with 7 medication categories (Table 1). These criteria were retrospectively applied on data of 448 advanced cancer patients referred to the palliative care service. These patients had a median survival of only 15 days. The authors noted very high numbers of patients that were still on gastric protectants (50%) and statins (97%). It was noted that antihypertensive agents and antidiabetics should be interpreted differently in advanced cancer patients than in other patients, since higher values of blood pressure or blood glucose can be acceptable in this population, if asymptomatic.

#### Preventative medications

Todd et al. [15] carried out a systematic review into studies determining inappropriate use of preventive medication of 5 drug classes in patients with a life-limiting illness. In this review, they established a list with the most common inappropriate preventive medications used in this population (Table 1). Afterwards, they assessed the frequency of use of these medications in patients with advanced lung cancer in two hospitals (Table 2) [22]. No interventional study with the list of preventative medications has been published to date.

#### Medications for chronic diseases

Garfinkel et al. [16] described the medication use among end-stage cancer patients at the time of admission to home care hospice (Table 2). Medications that were included were used for chronic diseases. Drugs used for the oncological treatment were excluded. Appropriateness of medication was not assessed, but a stepwise recommendation for deprescribing was formulated based on the observation that at just 2 months before death, 23% of patients were still being treated with 12 or more drugs and 90% were still treated with 6 to 12 drugs.

### Tools non-specifically for cancer patients

#### Beers criteria

The Beers criteria is a frequently used method to deprescribe in the overall geriatric population. Since the first edition, it has been updated many times based on new insights and evidence [17]. The Beers criteria are commonly applied on the palliative cancer population although it has been developed for the geriatric population. Zhou et al. [29] used the Beers criteria to estimate the frequency of six specific classes of PIMs in chemotherapy order templates for hematologic malignancies (Table 2). In 45% of these order templates, medications considered as PIMs by the Beers criteria were found. The authors wanted to draw attention to these potential risks. However, it could also be considered that the Beers criteria are not a perfect match to deprescribe in the cancer population. Karuturi et al. [23] applied the Beers criteria combined with drugs to avoid in the elderly (DAE) to identify PIM use in a cohort of older patients with stage II/III breast and colorectal cancer (Table 2). Also the frequency of these PIMs was evaluated at different time points. The use of PIM was lower at 3–6 months following initiation of chemotherapy when compared to baseline. Hong et al. [25] used the Beers criteria of 2015 to assess the frequency of PIMs in a population of geriatric patients with cancer undergoing first-line palliative chemotherapy (Table 2). However, medications typically used during chemotherapy (e.g., medications for nausea) were excluded. The authors state that modifying of the Beers criteria can be needed for the cancer population since several supportive drugs used during chemotherapy are considered PIMs by the Beers criteria. Nightingale et al. [26] combined 3 deprescribing guidelines: STOPP criteria, DAE, and the Beers criteria. The authors evaluated the frequency of PIMs in a population of ambulatory older adults with cancer. They divided the population based on the use of complementary and alternative medication (CAM). Herbal medications, minerals, or other dietary supplements, excluding vitamins, were considered as CAMs. The prevalence of the use of CAMs was 26.5%. In 2017, the same research group assessed appropriateness of medication use by the Beers criteria in patients who received a comprehensive geriatric oncology assessment and received a pharmacist-led individualized medication assessment and planning (iMAP) intervention [28]. They enrolled 41 patients in their study and identified medication-related problems in 95%. The pharmacists’ interventions reduced the number of medication-related problems by 45.5%. Flood et al. [27] finally applied the Beers criteria to 47 hospitalized older adult cancer patients referred to the acute care for elders unit (Table 1). The frequency of PIMs was determined on admission, and recommendations for deprescribing were made in 28% of patients. Again, the most frequent PIMs identified according to the Beers criteria were in fact drugs used as supportive care during cancer treatment.

#### STOPP criteria

STOPP criteria have been shown to improve inappropriate medication use in the elderly when applied during hospitalization [18]. Inappropriate medication use is associated with the occurrence of adverse drug events (ADEs) and an intervention using the STOPP criteria can reduce ADEs in older hospitalized patients. Karuturi et al. [24] used these criteria for estimating the use of PIMs in patients with stage II/III breast and colorectal cancer receiving chemotherapy, but found no statistically significant associations by the number of PIMs and clinical outcomes. As described above, Nightingale et al. [26] also used the STOPP criteria combined with 2 other guidelines.

#### Medication appropriateness index

The medication appropriateness index (MAI) is a questionnaire to assess futility of the drugs used (Table 1). It can be used to determine why the discontinued drug was inappropriate. Domingues et al. [19] applied a modified version of MAI for cancer patients at the time of palliative care transition in a prospective study (Table 2). They included 71 patients and found polypharmacy in 85% of cases. Using the MAI, 28% of drugs used could be suspended.

## Discussion

In this review, six deprescribing tools or guidelines specifically for cancer patients and three deprescribing tools or guidelines not specifically designed for cancer patients were identified.

On average, far more literature on the incidence of PIMs is available, than on methods for reducing them. This has also been noted in a large Delphi study evaluating better drug use in advanced disease [30]. The potential positive effects of deprescribing are multifold and have been demonstrated in several independent studies [2, 5–9]. Hence, to implement the concept of deprescribing in routine clinical care of palliative cancer patients, guidelines or consensus statements will be of great value.

Of the deprescribing tools we found for cancer patients, only the OncPal tool has been externally validated and has been applied by more than one research group [10, 20, 21]. The OncPal tool gives guidance for the majority of preventative drugs used, but lacks guidance on drugs used for thrombosis prophylaxis. In addition, no guidance on which drugs can be stopped in one step and which drugs should be tapered is given. Furthermore, OncPal has not been validated for palliative cancer patients with a life expectancy of more than 6 months whereas this population could also benefit from deprescribing. Of the deprescribing tools for the general population, the Beers criteria for geriatric patients have been studied most extensively and have also proven to be of value in cancer patients. Moreover, they are often updated and reviewed. However, some of the drugs that Beers criteria identify as being stoppable may actually be warranted in the specific group of cancer patients, either because they are part of supportive regimens for palliative chemotherapy, or because the risk–benefit is different in a palliative cancer patient when compared to a non-cancer geriatric patient. An example of the latter is the use of benzodiazepines to treat anxiety, which can be considered suboptimal due to tolerance, dependence, and fall-risk issues in general, but which in fact may be a good option if the expected end-of-life is too near to install other anxiety-reducing strategies.

Overall, we consider the OncPal guideline a good option and would recommend adding more drug classes, such as anticoagulants, to the tool. This population is at higher risk for deep vein thrombosis (DVT) due to their cancer or comorbidities, such as atrial fibrillation, and they are therefore more likely to use anticoagulants [31, 32]. However, limited clinical evidence is available regarding the prolonged use of anticoagulants as prophylaxis for palliative cancer patients. Furthermore, the frequently used anticoagulants coumarins can be a burden due to the regularly need of taking blood for INR monitoring. Unfortunately, some tumor types are associated with an increased risk of bleeding complications and increased risk for DVT [32]. Therefore, for each individual patient, the risk–benefit ratio should be assessed considering the use of anticoagulants. The OncPal guideline could well be combined with the stepwise methods described by Gonçalves [11] and Sharma et al. [12], because these methods incorporate a patient individualized approach. Finally, guidance from the Beers criteria on how to deprescribe (via stopping or via tapering) could well be a valuable addition to OncPal.

Future studies are needed to study the difference in clinical outcomes and quality of life in the palliative cancer population of the tools and guidelines identified in this review.

In conclusion, the tools, guidelines, and recommendations compiled in this review can help to support overdue standardization efforts to safely and effectively minimize unnecessary polypharmacy in palliative cancer patients.

## Data Availability

N/A.

## Code availability

N/A.
